# Multilevel Modelling with Spatial Interaction Effects with Application to an Emerging Land Market in Beijing, China

**DOI:** 10.1371/journal.pone.0130761

**Published:** 2015-06-18

**Authors:** Guanpeng Dong, Richard Harris, Kelvyn Jones, Jianhui Yu

**Affiliations:** 1 Sheffield Methods Institute, Faculty of Social Sciences, The University of Sheffield, Interdisciplinary Centre of the Social Sciences (ICOSS), 219 Portobello, Sheffield, S1 4DP, United Kingdom; 2 School of Geographical Sciences, University of Bristol, University Road, Bristol, BS8 1SS, United Kingdom; 3 Key Laboratory of Regional Sustainable Development Modelling, Institute of Geographical Sciences and Natural Resources Research, CAS, Beijing, China; Peking UIniversity, CHINA

## Abstract

This paper develops a methodology for extending multilevel modelling to incorporate spatial interaction effects. The motivation is that classic multilevel models are not specifically spatial. Lower level units may be nested into higher level ones based on a geographical hierarchy (or a membership structure—for example, census zones into regions) but the actual locations of the units and the distances between them are not directly considered: what matters is the groupings but not how close together any two units are within those groupings. As a consequence, spatial interaction effects are neither modelled nor measured, confounding group effects (understood as some sort of contextual effect that acts ‘top down’ upon members of a group) with proximity effects (some sort of joint dependency that emerges between neighbours). To deal with this, we incorporate spatial simultaneous autoregressive processes into both the outcome variable and the higher level residuals. To assess the performance of the proposed method and the classic multilevel model, a series of Monte Carlo simulations are conducted. The results show that the proposed method performs well in retrieving the true model parameters whereas the classic multilevel model provides biased and inefficient parameter estimation in the presence of spatial interactions. An important implication of the study is to be cautious of an apparent neighbourhood effect in terms of both its magnitude and statistical significance if spatial interaction effects at a lower level are suspected. Applying the new approach to a two-level land price data set for Beijing, China, we find significant spatial interactions at both the land parcel and district levels.

## Introduction

Many geographical data sets have multilevel structures—for example, houses nested into districts into regions in an urban housing market, or cities nested into regions, that are further nested into countries. Using the language of the multilevel modelling literature, the finer spatial scale at which an outcome variable is measured is termed the lower level whereas the more aggregate spatial scale is called the higher level. The multilevel modelling anticipates both differences between the higher level units and correlations within those units. The correlations within units are expected because their members are assumed to be affected by the same aggregate effects. The within group correlation is usually termed group dependence. The existence of group dependence among lower level units violates the classic assumption of independence in a standard regression analysis, raising the risk of inefficient model estimation and incorrect inference [1].

In a geographical setting, group effects are often termed place, contextual or neighbourhood effects. Multilevel models (MLM) have been widely applied to model and identify the existence of these effects [2]. Contextual effects refer to the idea that local contexts (represented by the higher level units) could affect the outcomes of lower level units belonging to them even conditioning on both higher and lower level covariates. For example, in geographical studies of health, the local context where individuals live has been found to make a difference in terms of a wide range of people’s health outcomes—people with nearly identical personal attributes and socio-economic characteristics but who live in different areas can have divergent health conditions [3].

However, in a geographical setting, it is not only the contextual effects that create the group dependence. Spatial interactions between the lower level units that are closely co-located (interactions that are termed spatial spillover effects in the spatial econometrics literature) can contribute to the correlations amongst group members. This issue has been rarely discussed in the multilevel modelling literature. Indeed, in its most common specification, the MLM is not really an explicitly spatial form of analysis at all. This can be seen by the absence of a spatial weights matrix giving the proximity between units, whether it is contiguity based, or based on Euclidean distance defining the ‘*n*’ nearest neighbours. No geographical information as such is passed to the MLM beyond the grouping of lower level units into higher level ones.

For geographically referenced data sets, we might expect that the outcome at a particular location would be influenced by its surrounding locations with the intensity of this influence being determined by the geographic proximity. This spatial interaction effect is nonetheless difficult to model using classic multilevel models. This is because, at the lower level, the correlation structure among observations is defined as discontinuous, bounded by the geographical boundaries of the higher level units. Consequently, two lower level units located either side of a boundary are assumed to be independent even though they are in close geographical proximity. Secondly, the (unobserved) contextual effects (which are the higher level residuals) are presumed to be independent. Consequently, any spatial proximity effects at the higher level are not taken into account. Whereas both contextual and spatial proximity effects may affect the outcome of interest, the MLM can only consider the first effect, which may be confounded with the second and incorrectly estimated as a result.

Based on these observations, some efforts have been made to distinguish between the contextual effect and spatial interaction effects in the spatial econometrics literature. In approaches developed in [4] and [5], the higher level random effect has been incorporated into a classic spatial econometric model (spatial lag models) so to better estimate the spatial interaction effect at the lower level. However, possible spatial interactions among higher level units are left unmodelled. On the other hand, spatial interaction effects are exclusively considered at a higher level in models proposed in [6] and [7]. Recently, a general hierarchical spatial autoregressive modelling framework for geographically hierarchical data has been developed in [8], in which both spatial interaction effects at each level of a spatial hierarchy and the contextual effect can simultaneously be modelled. This paper further demonstrates this approach as an appropriate way to incorporate spatial interactions into the classic MLM framework if simultaneity or endogeneity is suspected to be inherent in the outcome under study. In addition, the performance of this approach under various scenarios is assessed through extensive Monte Carlo simulations.

The remainder of this paper is organised as follows. First, principles of the classic MLM and existing efforts to incorporate spatial interaction effects into the MLM briefly are reviewed. We then describe the approach developed in [8] and evaluate the validity of this approach against the MLM via a simulation study. Next, the proposed method is employed to investigate the land market in Beijing, China and the results are compared with that from a classic MLM.

## Materials and Methods

### Multilevel models and spatial effects

**Fig 1:**
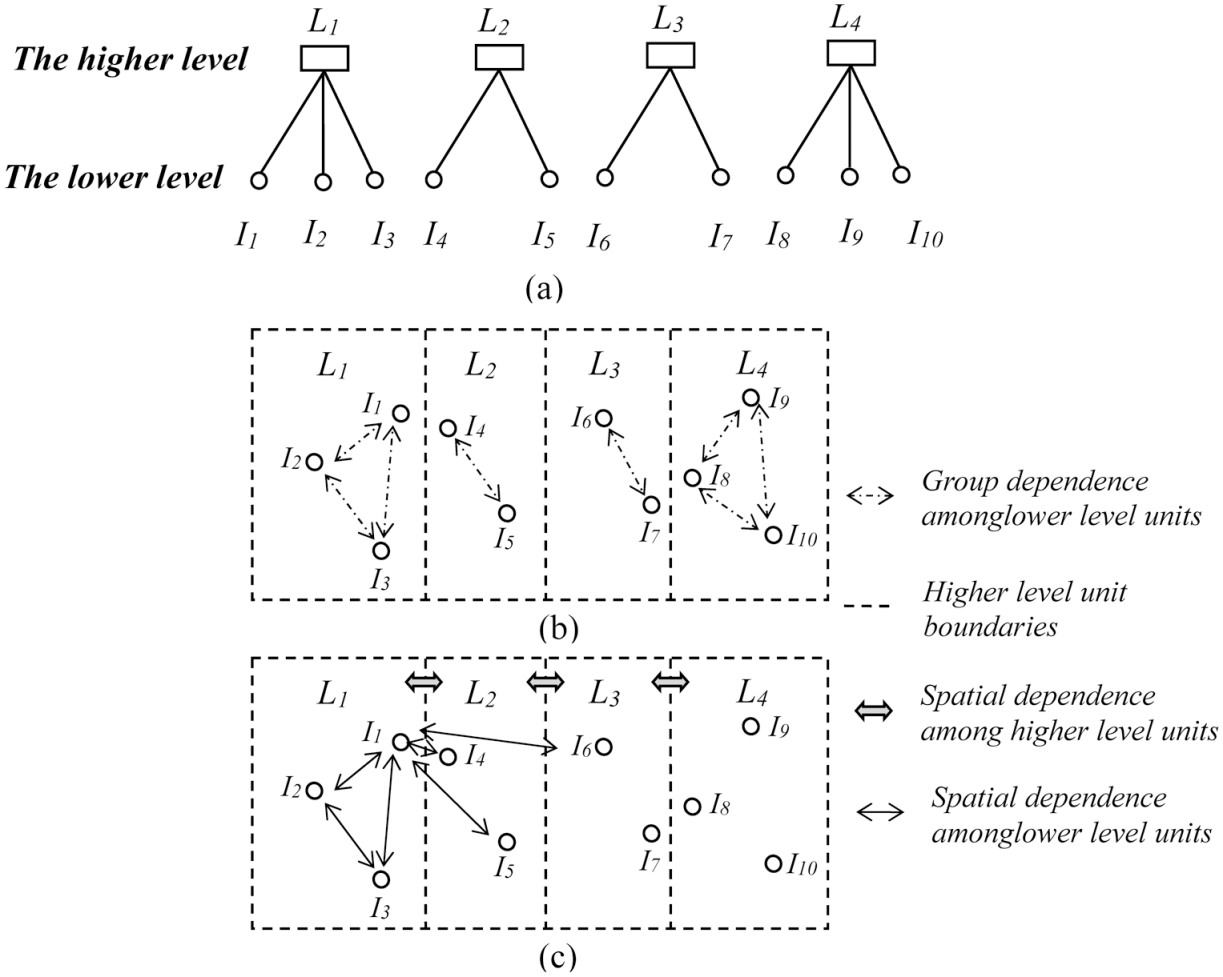
A two level data structure shown hierarchically and also as a map.

Consider a two level geographically hierarchical dataset where there are *N* lower level units nested into *J* higher level units that each contains *n*_*j*_ lower level units. A simplified graphical typology of this structure is shown in Fig 1a. There are four higher level units (*L*_*1*_ to *L*_*4*_) and ten lower level units (*I*_*1*_ to *I*_*10*_). Fig 1b) and 1c are the corresponding geographic representations of Fig 1a.

Following [1], a random intercept MLM is specified as,

yij=β0+xijTβ+xjTγ+uj+εijvar(εij)=σe2;var(uj)=σu2cov(εij,uj)=0;cov(εijεi'j')=0ifj≠j' (1)

where *i* and *j* indicate the lower and higher levels, respectively, *y*_*ij*_ is the outcome variable, *x*_*ij*_ and *x*_*j*_ refer to the lower and higher level independent variables, ***β*** and ***γ*** are the corresponding regression coefficient vectors to estimate (***β*** at the lower level, ***γ*** at the higher level), *ε*_*ij*_ is the lower level residuals, assumed to follow an independent normal distribution, N (0, *σ*_e_^2^), and *u*_*j*_ are the higher level residuals also assumed to follow an independent normal distribution, N (0, *σ*_*u*_^*2*^). It is *u*_*j*_ that, in a geographical setting, are taken to measure unexplained contextual effects. The covariance between lower level units in the same higher level unit is cov(*y*_*ij*_, *y*_*i’j*_) = cov(*u*_*j*_ + *ε*_*ij*_, *u*_*j*_ + *ε*_*i’j*_) = *σ*_*u*_^2^ where *i*’ denotes different lower level units in the *j*th higher level unit. The non-zero covariance indicates the group dependence at the lower level. Moreover, the intensity of the group dependence is quantified by cov(*y*_*ij*_, *y*_*i’j*_) /var(*y*_*ij*_) = *σ*_*u*_^2^/(*σ*_*u*_^2^+ *σ*_*e*_^2^), which is known as the variance partitioning coefficient (VPC) [1,2]. As illustrated in Fig 1b, there are correlations among the lower level units in the same higher level unit and the strength of these correlations is *σ*_*u*_^*2*^/ (*σ*_*u*_^*2*^+ *σ*_*e*_^*2*^) for each higher level unit.

The possibility of a proximity based spatial interaction effect is illustrated in Fig 1c. At the lower level, consider the example of *I*_*1*_ in *L*_*1*_. Rather than just assuming it is correlated with *I*_*2*_ and *I*_*3*_ as in the MLM, it could also interact with *I*_*4*_, *I*_*5*_ and *I*_*6*_ because they are nearby. In contrast, because *I*_*7*_, *I*_*8*_, *I*_*9*_ and *I*_*10*_ are far from *I*_*1*_, we might assume their spatial proximity effect on *I*_*1*_ will reduce to zero. With respect to the magnitude of the interactions, it seems that the intensity of correlation between *I*_*1*_ and *I*_*2*_ should be larger than that between *I*_*1*_ and *I*_*3*_ because the first pair has less of a geographical separation. Moreover, at the higher level, *L*_*1*_, *L*_*2*_, *L*_*3*_ and *L*_*4*_ are also potentially interacting with each other. Note, again, that a classic MLM approach does not allow for proximity effects other than the grouping of lower level units into higher level ones.

### Existing work on multilevel models with spatial interactions

In the geographies of health literature, some have recognised both the contextual effects and the spatial interaction effects and have modified the classic MLM accordingly. Langford et al. (1999) add an additional lower level spatial random variable *v*_*i*_ into Eq (1) [9]. It is a weighted sum of a set of independent random effects v* from neighbouring units such that,

$$v_{i}=\sum_{k\neq i}w_{ik}v_{k}^{*}$$ (2)

where *w*_*ik*_ are entries of a row-normalised spatial weights matrix *W* at the lower level, defined by geographic distances. The random effects v*are distributed as N (0, *σ*_*v*_^*2*^). Through this adjustment to the MLM, spatial interaction effects at the lower level are modelled. This can be seen from the conditional covariance matrix of *y* [9],

$$cov(y\text{|}x,\beta ,\gamma ,u)=\sigma_{e}^{2}I_{N}+\sigma_{\varepsilon ,v*}(W+W^{T})+\sigma_{v*}^{2}WW^{T}$$ (3)

where *σ*_ε,v*_ is the covariance between *ε* and v*, *x* includes both lower and higher level covariates and *I*_*N*_ is an identity matrix with order of *N*.

In a similar vein, Browne et al. (2001) propose a multiple-classification, multiple-membership, multilevel model (MMMC) based on their formal definition of classifications to tackle spatial dependence [10]. The idea is to consider the neighbours for each lower level unit as an additional multiple membership classification. Using their notation, a MLM with spatial interactions can be specified as,

yi=β0+xiTβ+uContext(i)(2)+∑k∈Neighbour(i)wikvk*(3)+εiuContext(i)(2)~N(0,σu2),vk*(3)~N(0,σv2),εi~N(0,σe2) (4)

where the classification function Context(*i*) assigns the *i*th lower level unit to its higher level unit, and Neighbour(*i*) identifies its set of neighbours. The term *u*_Context(i)_^(2)^ is the higher level random effects and *v*_k_*^(3)^ is the neighbourhood level random effects. The departure from the modification in [9] is the assumption of independence between v* and *ε*. Therefore, *σ*_ε,v*_ in Eq (4) is zero. This technique has been employed to investigate the impact of the network dependence on students' educational attainment [11]. Other modifications of the MLM directly conceptualise the random effect *v*_*i*_ in Eq (2) as a conditional autoregressive process or a Gaussian spatial process [10,12,13].

To summarise, the above modifications to the classic MLM take a conditional approach. That is, an assumption of conditional independence of the outcome under study is imposed, indicating that changing characteristics of covariates (*x*_*ij*_) will not influence outcomes of nearby units. Therefore, these methods are not so useful when substantial spatial interaction effects are expected, when the outcome at one location is directly related to the outcomes at surrounding locations. Possible simultaneity or endogeneity inherent in the outcome variable under study cannot be properly modelled using the above adjustment to the MLM. Moreover, spatial interaction effects among the higher level units are generally not taken into account.

### Proposed multilevel models with spatial interaction effects

With the limitations of the existing work on incorporating spatial interaction effects into the MLM in mind, our proposal is to integrate simultaneous autoregressive (SAR) processes for both the response variable and higher level residuals into the classic MLM. The SAR process has been extensively discussed in the spatial econometrics literature and widely used in geographical research [14,15,16,17,18]. A key characteristic of a SAR process is that it allows the observed value at a particular location to be directly dependent on the values observed at surrounding locations (or lagged y), in this way allowing for an interaction (or spatial spillover) effect to be both specified and measured. Following [8], the MLM with spatial interaction effects is specified as,

$$\begin{array}{c} y=\rho Wy+X\beta +Z\gamma +\Delta \theta +\varepsilon \\ \theta =\lambda M\theta +u\\ \Delta =\left[\begin{array}{cccc} l_{1} & 0 & \cdots & 0\\ 0 & l_{2} & \cdots & 0\\ \cdots & \cdots & \cdots & \cdots \\ 0 & 0 & \cdots & l_{J} \end{array}\right] \end{array}$$ (5)

where *y* is an *N* by 1 column vector of outcome variable values; *X*, an *N* by *K* matrix, denoting the lower level covariates; ***β*** is a *K* by 1 vector of regression coefficients to estimate; *Z* is an *N* by *P* matrix consisting of higher level variables; ***γ*** is the corresponding *P* by 1 coefficient vector; *W* is the spatial weights matrix among lower level units as in Eq (2); *ρ* is a spatial autoregressive parameter indicating the strength of spatial interactions at the lower level; Δ is an *N* by *J* block diagonal design matrix with column vectors of ones; and *ε* denotes the lower level residuals, which are distributed as N (0, *σ*_*e*_^*2*^).

The *J* by 1 vector of higher level residuals ***θ***[*θ*_*1*_, *θ*_*2*_,…, *θ*_*J*_] represent the random contextual effects. The residuals *u* are distributed as N (0, *σ*_*u*_^*2*^) and are assumed to be independent of *ε*. Like *W*, *M* is also a row-normalized spatial weight matrix but at the higher level. The parameter *λ* measures the intensity of spatial interactions at the higher level. Specified by a SAR process, the covariance matrix for ***θ*** is cov(***θ***) = $\sigma_{u}^{2}{\left(B^{'}B\right)}^{-1}$ where *B* = *I*_*J*_ *-λM*. The distribution of ***θ*** therefore is multivariate normal, ***θ*** ~ N (***0***, $\sigma_{u}^{2}{\left(B^{'}B\right)}^{-1}$).

From Eq (5) we see that the conditional expectation of *y* is,

$$E(y\text{|}X\beta ,Z\gamma )={\left(I_{N}-\rho W\right)}^{-1}(X\beta +Z\gamma )$$ (6)

which means that changing the values of the covariates at one location in the proposed model will affect not only its own outcomes but also outcomes at other locations because of (*I*_*N*_–*ρW*)^-1^. Also, the substantial spatial interaction effect can be seen from the marginal effects for each covariate. For example, the marginal effects for a lower level variable *x*_*k*_ are,

$$\begin{array}{l} \partial y/\partial x_{k}={\left(I_{N}-\rho \text{W}\right)}^{-1}\;I_{N}\beta_{k}=S_{k}\left(W\right)I_{N}\beta_{k}\\ S_{k}\left(W\right)={\left(I_{N}-\rho \text{W}\right)}^{-1}=\left(I_{N}+\rho \text{W}+\rho^{2}{\text{W}}^{2}+\ldots \right) \end{array}$$ (7)

which is the same as for a standard spatial SAR regression model because the contextual effects ***θ*** are assumed to be independent of *X*.

The proposed model is, therefore, hierarchical—allowing for parameter estimates at two levels. It also is spatial—estimating a spatial interaction effect and at two levels. The model is implemented via a Bayesian Markov Chain Monte Carlo (MCMC) simulation approach. Details on full conditional distributions for each parameter are provided in [8]. The MCMC sampler for implementing the proposed method is coded using the R language in the subsequent analyses (see S1 Appendix).

### Simulation study

In this section, a series of Monte Carlo simulations are conducted to evaluate the performance of the proposed methodology and to show how the issue of spatial interaction deteriorates the estimation from a MLM. The data generating process follows a MLM with spatial interaction effects (Eq (5)). The experiment includes 20 scenarios based on different combinations of spatial autoregressive parameters *ρ* [0, 0.2, 0.4, 0.6, 0.8] and *λ* [0.4, 0.6, 0.8, 0.9].

### Data generation

**Fig 2:**
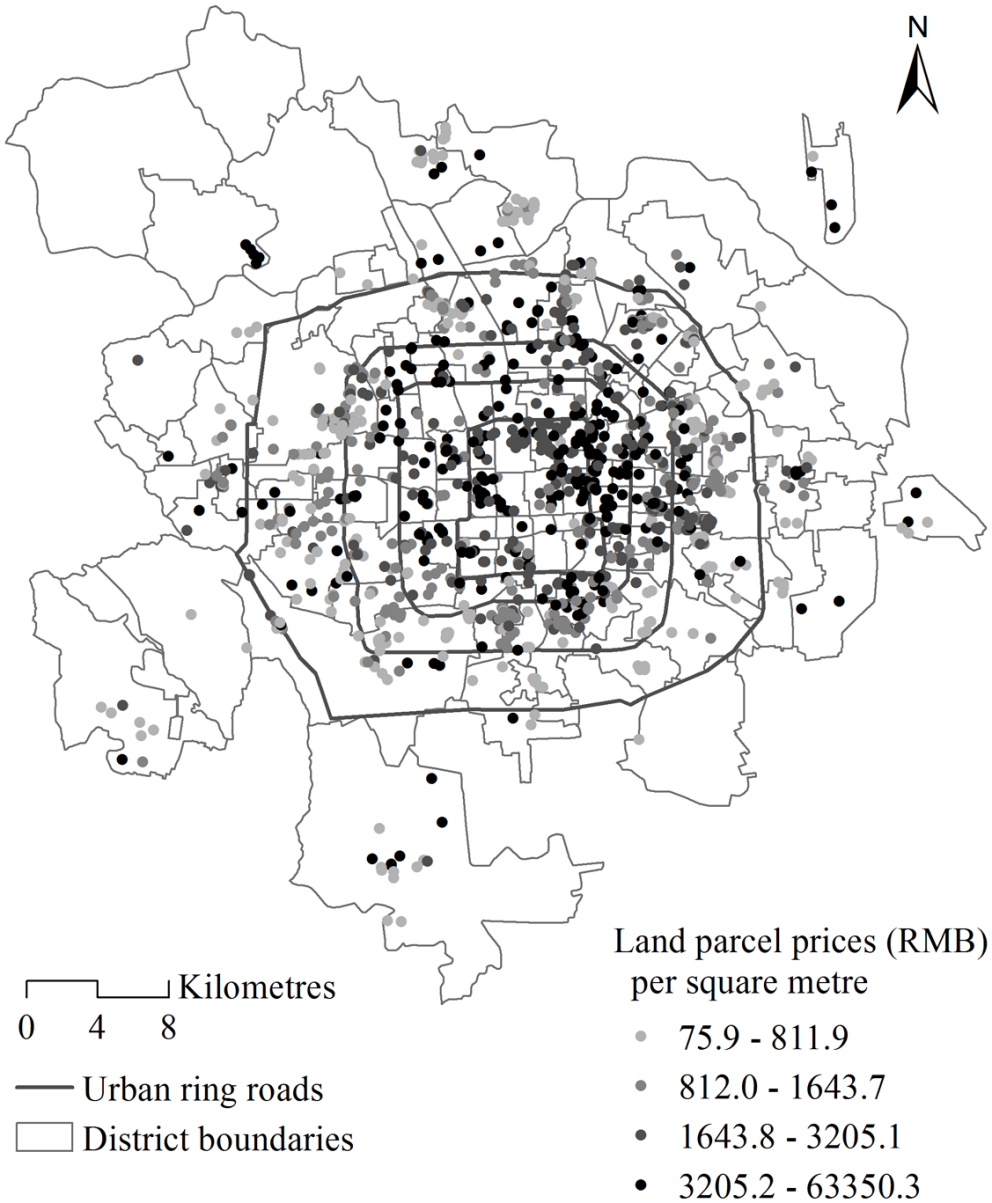
The price per area of residential land parcels leased between 2003 and 2009 within the study area of Beijing, China.

The spatial structure used is based on the real-world geography of the residential land parcels dataset in Beijing, China. There are 1117 land parcels (the lower level units) situated into 111 districts (*Jiedao*, the higher level units) in Beijing and we have both the geographic coordinates of those land parcels and the boundaries of the districts (Fig 2). A detailed description of the data is provided later.

The spatial weights matrix at the land parcel level *W* is specified as,

$$W_{ij}=\{\begin{array}{c} exp\left(-\left(d_{ij}{}^{2}\right)/d^{2}\;\right)\;if\;d_{ij}\leq d\\ 0\;\;if\;d_{ij}>d \end{array}$$ (8)

,which is an exponentially decay function where *d* is the distance threshold, set to 1.5km. This is the distance at which the correlations between land prices become negligible and was determined through an exploratory analysis using variograms [19,20]. In the following empirical land price model, we also conducted a sensitivity analysis using different threshold distances such as 2km and 2.5km; the model estimation results were similar. The Euclidian distance between land parcels is *d*_*ij*_. The district level spatial weight matrix *M* is based on the contiguity of the 111 districts. Both *W* and *M* are row-normalized in the subsequent analyses.

We generate four covariates: an intercept term, two land parcel level covariates (*X*_*1*_ and *X*_*2*_) and a district level covariate (*X*_*3*_). Each of these variables is generated from a standard Normal distribution, N(0,1). The corresponding regression coefficients ***β*** [*Intercept*, ***β***_1_, ***β***_2_, ***β***_3_] are set to [2,–2,3,1]. There are two steps to generate the outcome variable *Y*. First, the dependent district effects are generated using:

$$\theta ={\left(I-\lambda M\right)}^{-1}u,\;u\;\sim \;\text{N}(0,\sigma_{u}^{2})$$ (9)

where the variance of the higher level residuals, *σ*_*u*_^2^, is set to 0.4. The higher level spatial interaction term *λ* is sequentially set to [0.4, 0.6, 0.8, 0.9], indicating increasing intensity of spatial interactions at the higher level. The second step is to generate *Y* by using:

$$Y={\left(I-\rho \text{W}\right)}^{-1}(\text{X}\beta +\Delta \theta +\varepsilon ),\;\varepsilon \;\sim \;\text{N}(0,\sigma_{e}^{2})$$ (10)

where *X* = [**1**, *X*_*1*_, *X*_*2*_, *X*_*3*_], the variance of the lower level residuals, *σ*_*e*_^2^ is set to 0.8 and the lower level spatial interaction term *ρ* to [0, 0.2, 0.4, 0.6, 0.8]. Therefore, we have 20 scenarios in total using different combinations of values of *ρ* and *λ*.

Given the above setup, 200 simulated data samples were generated for each scenario. For each data sample, estimates of the model parameters were obtained using classic MLM and with our spatial extensions. The relative performance of these two models are assessed by the bias and root-mean-square error (RMSE) of the regression coefficients ***β***, two spatial autoregressive parameters (*ρ* and *λ*) and two variance parameters (*σ*_*e*_^2^ and *σ*_*u*_^2^), presented as a percentage of their true values [21]. For each data replication, the inference of the proposed method is based on 10000 draws with the first 5000 discarded to allow the MCMC sampler to converge. Diffuse or quite non-informative priors were employed for model parameters, ***β***, *ρ*, *λ*, *σ*_*e*_^2^ and *σ*_*u*_^2^ while the initial values were draw randomly from their corresponding distributions.

## Results and Discussions

### Simulation results

**Fig 3:**
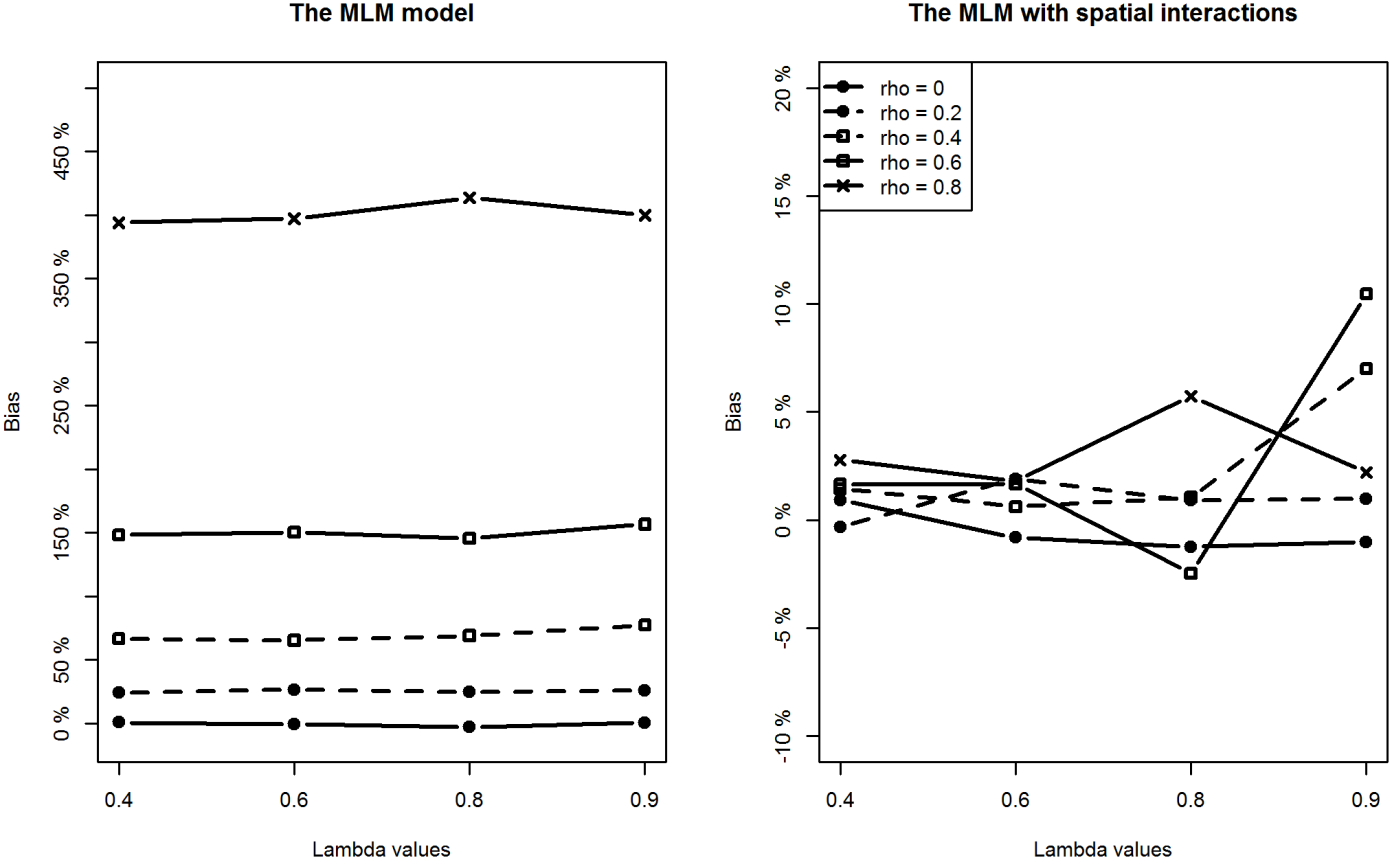
Comparing estimation results of the intercept term between classic MLM and the MLM with spatial interactions (Note the different y-scales).

**Fig 4:**
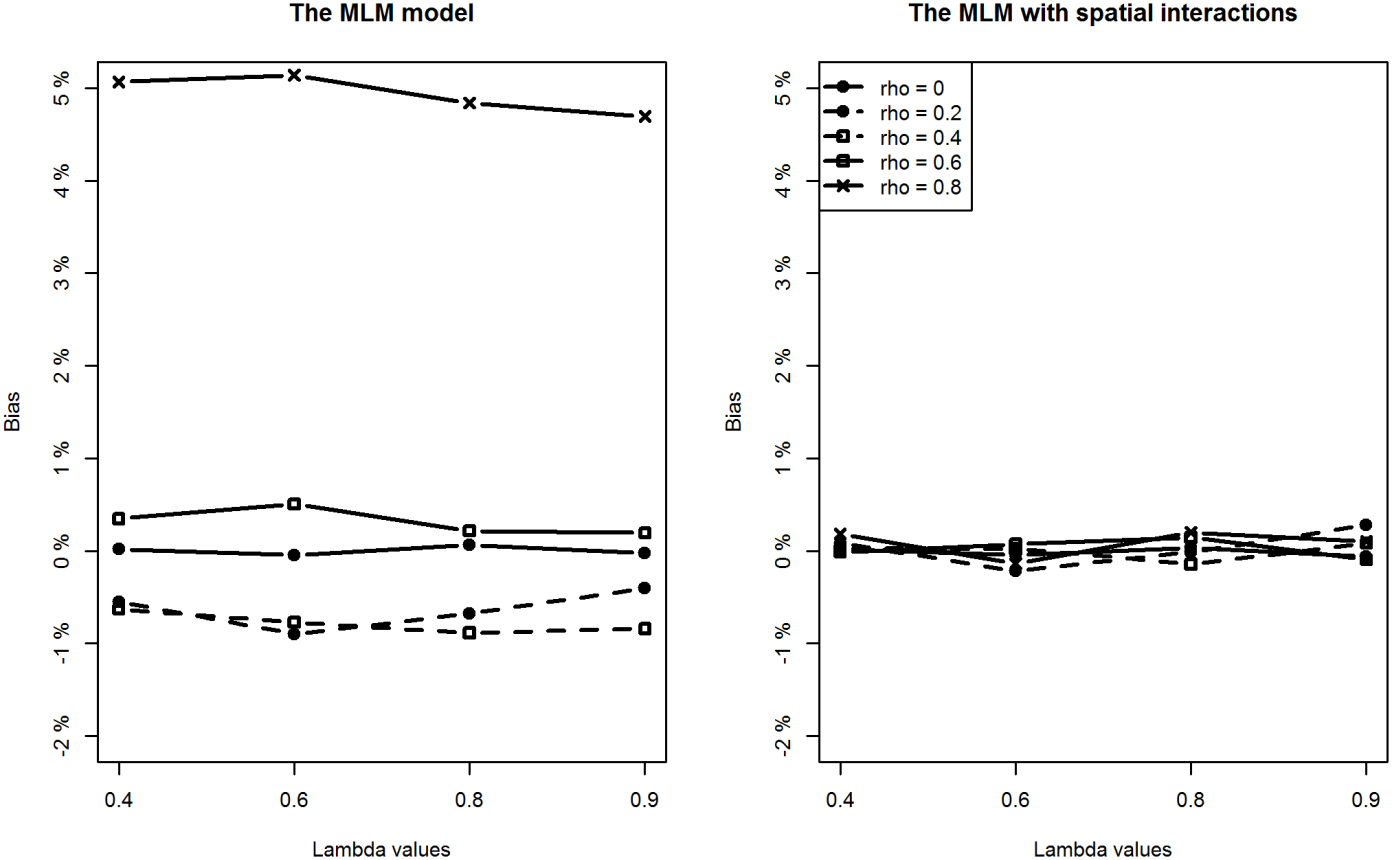
Comparing estimation results of *β*_1_ between classic MLM and the MLM with spatial interactions.

**Fig 5:**
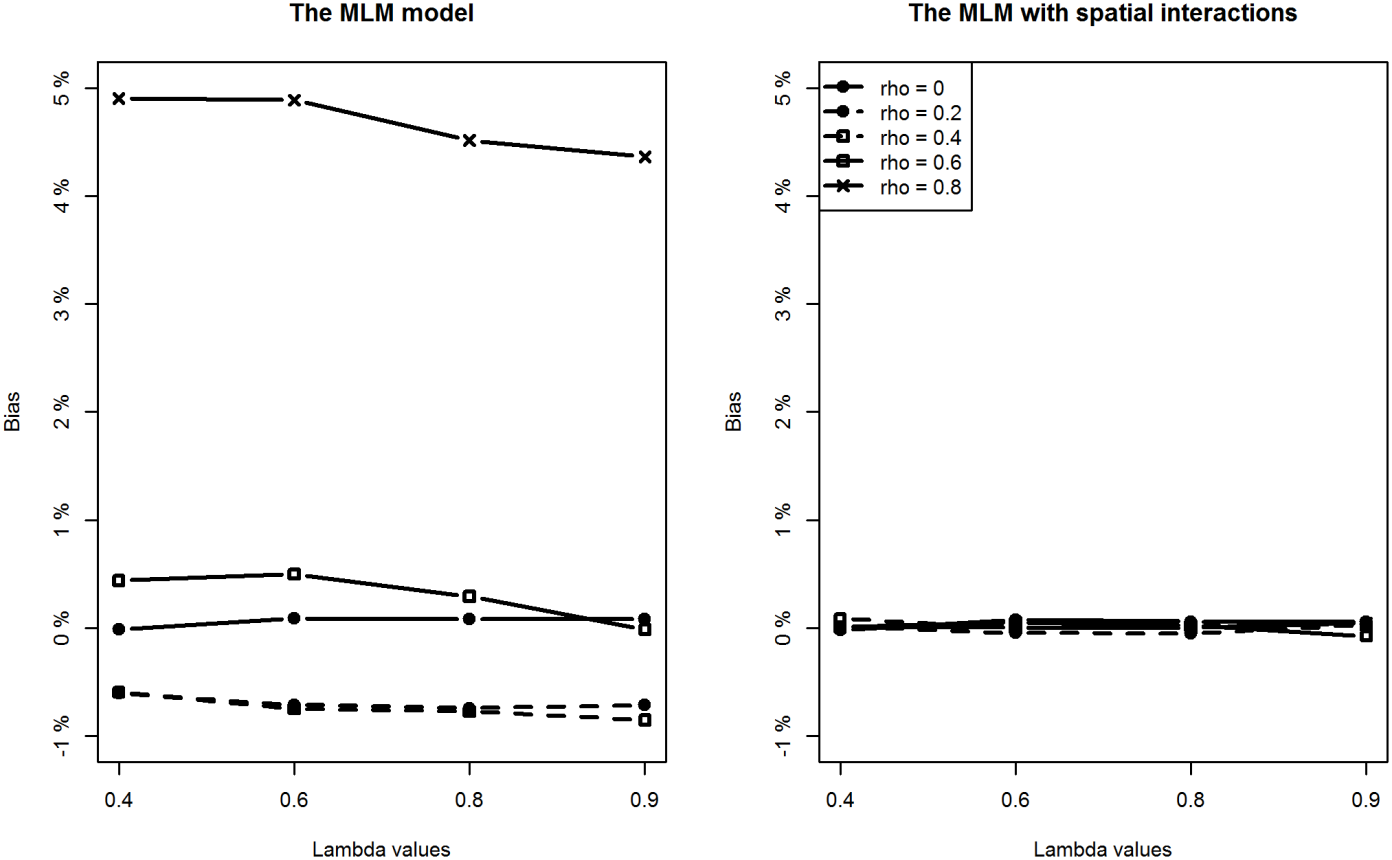
Comparing estimation results of *β*_2_ between classic MLM and the MLM with spatial interactions.

**Fig 6:**
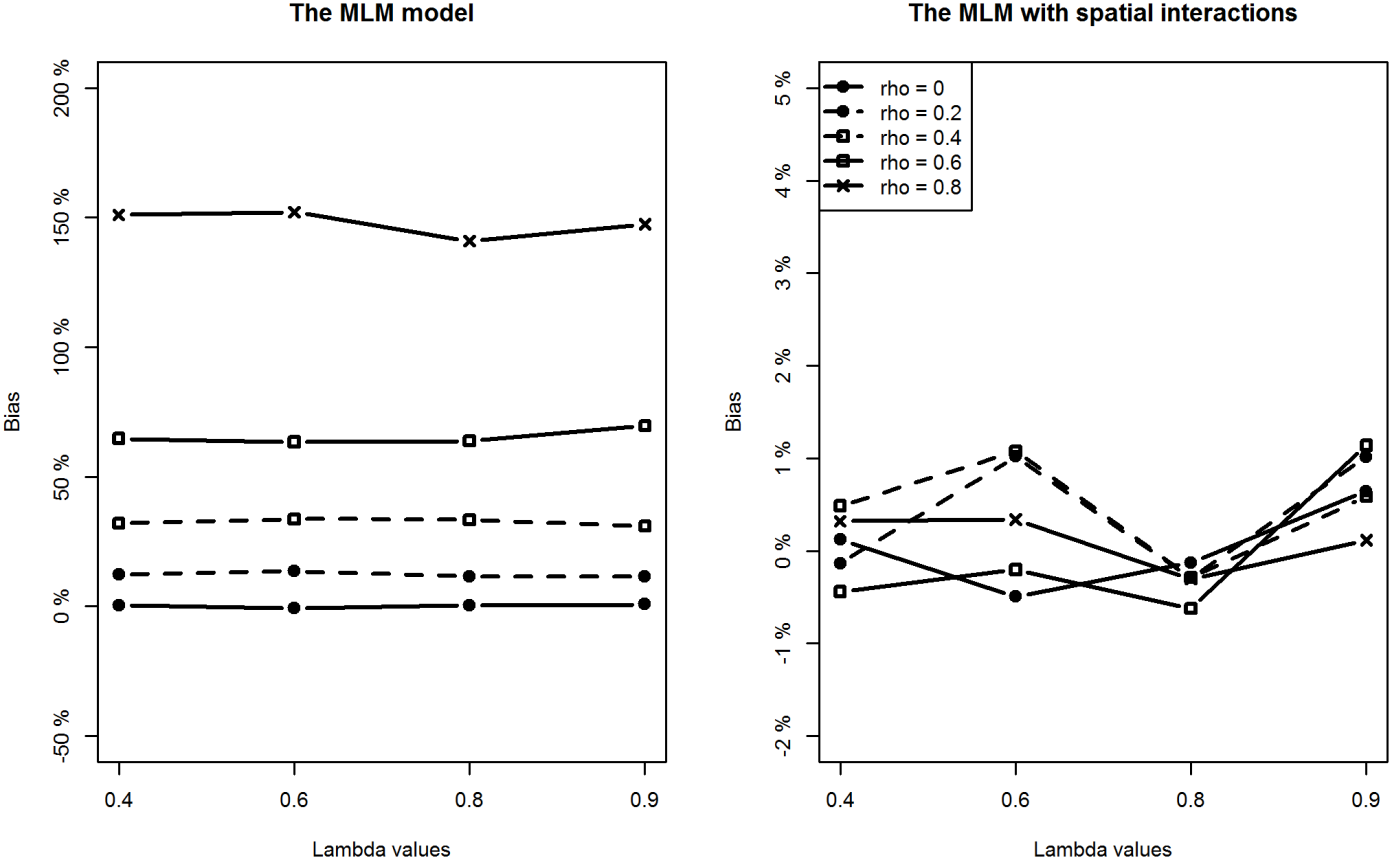
Comparing estimation results of *β*_3_ between classic MLM and the MLM with spatial interactions (Note the different y-scales).

With respect to the estimation of regression coefficients, the relative bias of the proposed model is negligible in most of the scenarios. In only a few scenarios, the estimation bias of the intercept term seems slightly large, reaching about 10% (still much smaller than the bias from the classic MLM model). In contrast, the situation is more complicated for the MLM. For lower level covariates (*X*_*1*_ and *X*_*2*_), the MLM provides slightly larger biased estimates when compared to the proposed method in all scenarios (Figs 4 and 5). However, of particular note are the estimates for the intercept and the higher level covariate effect, which are highly biased when the spatial interaction effect at the lower level is large. For example, when *ρ* is above 0.4, regardless of the intensity of spatial interaction effects at a higher level, estimation biases for the intercept term and the higher level covariate are very noticeable (Figs 3 and 6).

Additionally, we can see that estimation biases of regression coefficients are positively related to the strength of spatial interactions at a lower level. Recall that in many application studies, higher level covariate effects are interpreted as (observable) contextual or neighbourhood effects. An important implication of this simulation study is to be cautious of the identified neighbourhood effect in terms of both its magnitude and statistical significance if spatial interaction effects at a lower level are suspected.

As for the estimation precision indicated by the RMSE values for each covariate, we see the proposed method provides consistently more precise estimates for all the regression coefficients than the MLM does in most of the scenarios. The better performance of the proposed methodology is expected. We have, after all, generated the data using the proposed model. Nevertheless, the simulation serves to demonstrate an important point: if there are spatial interaction effects at the lower and higher levels, the MLM appears to produce biased and imprecise estimates for the regression coefficients.

As for the spatial autoregressive parameters (which can be estimated only with the proposed methodology, not the MLM), the estimation biases for *ρ* and *λ* are also quite small in all scenarios. The estimation bias of the higher level spatial autoregressive parameter *λ* is slightly larger than that of *ρ* in each scenario. This might be related to the relatively small number of higher level units [17, 22]. The lack of a sufficient effective sample size at the higher level also results in a larger RMSE for *λ* when compared to *ρ*. Overall, the proposed method performs well in retrieving the two true spatial autoregressive parameters.

**Fig 7:**
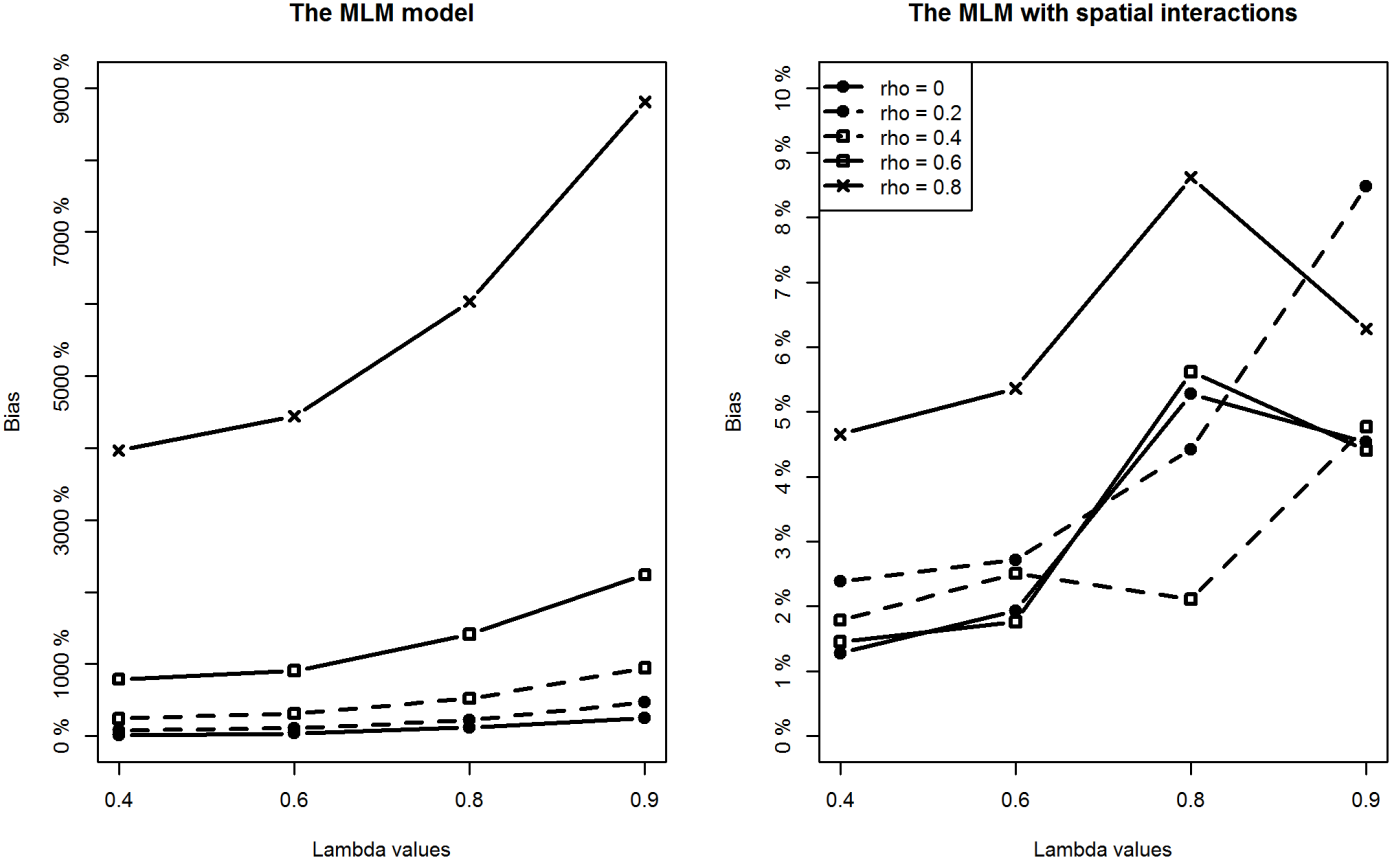
Comparing estimation results of *σ*_*u*_^2^ between classic MLM and the MLM with spatial interactions (Note the different y-scales).

With respect to the estimation of the two variance parameters, the proposed method performs well by providing accurate and precise estimates for both the higher level and lower level variances. In contrast, the MLM tends to overestimate their sizes because of unmodelled spatial interaction effects. The degree of the upward bias is striking for the higher level variance parameter *σ*_*u*_^2^ when the intensity of spatial interactions at the lower level is relatively large (Fig 7). For example, when *ρ* is about 0.6, the relative bias reaches about 800%. In addition, the degree of bias for *σ*_*u*_^2^ is positively related to the intensity of spatial interaction effects at both levels. This implies that in the presence of medium-to-strong degree of spatial interactions at a lower level, estimation of higher level variance from classic MLM is not reliable at all and nor is the usual variance partitioning coefficient (VPC) measure.

To access the accuracy of retrieving the (unobserved) contextual effects ***θ*** in the proposed method and in the MLM, for each of the 200 data replications in each scenario (20 in total) we calculate the correlation coefficients between the posterior means of ***θ*** from the two models and their true values. For all the 4000 (200×20) data replications, the mean correlation coefficient from the proposed method is 0.916 with an interquartile ranging from 0.888 to 0.949, indicating that the proposed method can retrieve the true higher level random effects accurately. In contrast, the mean correlation coefficient from the MLM is 0.781 with an interquartile ranging from 0.696 to 0.892.

### Land price modelling results

#### Data and variables

In this section, we apply the proposed methodology to analyse an emerging land market in Beijing based on all residential land parcels from 2003 to 2009 leased by the government. The outcome variable is the leasing price per square metre of each residential land parcel, adjusted by using the consumer price index (CPI). The land parcel level covariates used here are the land parcel size (Logarea) and the proximity of each land parcel to the nearest subway stations (LogDsubway), to elementary schools (LogDele), to green parks (LogDpark), to rivers (LogDriver) and to the central business district (LogDcbd). Year dummies (Year04—Year09) are also included in the model to control for fixed period effects, with the year 2003 as the baseline. The district level covariates are the population density of each district (Popden), the proportion of houses built before 1949 (Buildings1949), and the number of violent crimes taking place per 1000 people (Crimerate). The natural log transformation is applied to the proximity measures to mitigate the potential problem of heteroskedasticity. The land price data used here is in S1 Dataset and a brief summary of the complete set of variables, including their definition, mean and standard deviation is presented in Table 1.

**Table 1:** Summarising the Chinese land parcel and district data used in the analysis.

Variables	Definition	Mean	Std.Dev
***Dependent Variable***			
Logprice	Log of the land parcels' leasing price per square meter (RMB/m^2^)	7.414	1.029
***Lower level variables (land parcels)***			
Logarea	Log of the land parcel area (m^2^)	9.301	1.532
LogDcbd	The log of the distance between a land parcel and the CBD	8.956	0.675
LogDele	The log of the distance to the nearest elementary school	6.591	0.920
LogDpark	The log of the distance to the nearest park	7.774	0.704
LogDriver	The log of the distance to the nearest river	7.516	0.931
LogDsubway	The log of the distance to the nearest subway station	7.148	0.895
*Year dummies*	The year when the land parcel was leased		
***Higher level variables***			
Buildings1949	Percentage of buildings built before 1949 in each *Jiedao*	0.042	0.109
Crimerate	Number of reported serious crimes per 1000 people in each *Jiedao*	5.246	6.112
Popden	Population density in each *Jiedao* (1000 people/km^2^)	1.937	2.670

#### Estimation results

The estimation results from the proposed model and the MLM are provided in Table 2. We also examined the potential problem of multicollinearity using the variance inflation factor (vif) scores for the predictor variables. There is no evidence of such a problem with vif values of about 1.2 (much less than the normal thresholds for concern of five or ten and close to the ideal value of one).

**Table 2:** Regression coefficients estimation results from the land parcel price data set.

	MLM with spatial interaction effects				Classic MLM			
	Posterior mean	Std. Error	2.5%	97.5%	Posterior mean	Std. Error	2.5%	97.5%
Intercept	11.357*	0.958	9.448	13.248	13.627*	0.702	12.211	14.998
Logarea	-0.023	0.018	-0.059	0.013	-0.031	0.019	-0.068	0.006
LogDcbd	-0.362*	0.102	-0.576	-0.168	-0.373*	0.075	-0.520	-0.227
LogDsubway	-0.177*	0.042	-0.258	-0.097	-0.198*	0.042	-0.278	-0.115
LogDele	-0.015	0.039	-0.091	0.062	-0.054	0.038	-0.127	0.019
LogDpark	-0.148*	0.061	-0.266	-0.031	-0.245*	0.056	-0.359	-0.136
LogDriver	0.099*	0.036	0.031	0.168	0.137*	0.036	0.069	0.206
Popden	0.019	0.013	-0.007	0.044	0.029*	0.014	0.001	0.056
Buildings1949	-1.082*	0.518	-2.067	-0.050	-1.380*	0.426	-2.211	-0.544
Crimerate	0.001	0.008	-0.015	0.017	0.010	0.010	-0.010	0.029
Year04	-0.209*	0.056	-0.320	-0.104	-0.191*	0.056	-0.299	-0.083
Year05	-0.048	0.118	-0.281	0.188	-0.024	0.119	-0.259	0.216
Year06	-0.064	0.103	-0.272*	0.137	-0.077	0.105	-0.281	0.133
Year07	0.732*	0.114	0.507	0.955	0.736*	0.118	0.505	0.959
Year08	0.535*	0.127	0.288	0.775	0.564*	0.128	0.313	0.816
Year09	2.326*	0.217	1.904	2.747	2.187*	0.216	1.762	2.607
*ρ*	0.174*	0.036	0.104	0.246	NA	NA	NA	NA
*λ*	0.760*	0.145	0.387	0.959	NA	NA	NA	NA
*σ* *_u_* ^2^	0.052*	0.021	0.020	0.102	0.125*	0.030	0.075	0.192
*σ* *_e_* ^2^	0.574*	0.025	0.525	0.624	0.579*	0.026	0.530	0.633

* denotes statistically significant at 95% credible level or above.

From the proposed method we see that the spatial autoregressive parameters both at the land parcel level (*ρ*) and the district level (*λ*) are statistically significant at the 95% credible level. This indicates the existence of spatial interactions among land parcels in the price formation process and that land parcel prices are impacted by effects from their own district (or immediate context) as well as by effects from surrounding districts. The latter cannot be measured by the classic MLM and so the two are confounded.

We also see that the MLM produces quite different estimates for most of the covariates when compared to the proposed method. Most noticeably, district population density exerts significant inference on land prices in the MLM while it does not do so in the proposed methodology. As with the results from the simulation study, the MLM significantly overestimates the district level variance *σ*_*u*_^2^: the 95% credible intervals for the estimates of *σ*_*u*_^2^ in the proposed method do not contain the mean estimate of *σ*_*u*_^2^ in the MLM. This is expected if some of the (unobserved) contextual effects are actually due to spatial interaction effects.

#### Interpreting the covariate effects

With respect to the sign of the covariate effects, most of them are as expected. From the estimation results of the proposed model in Table 2, the accessibility to city centre (LogDcbd) appears to have a significant impact on land prices, indicating the existence of negative land price gradients moving away from the city centre after China's market-oriented urban land reforms. This is in accordance with the classic urban land bid rent theories [23,24,25]. Increased proximity to nearest subway stations and to green parks tends to increase land prices, which indicates the importance of convenience (good transport accessibility for work or non-work activities) and environmental amenities in residents’ housing location choices in Beijing, China. Surprisingly, proximity to rivers is negatively associated with land prices, which might reflect the situation where most of the rivers in the urban areas of Beijing were severely polluted specifically before the Olympic Games in 2008 [25].

For the district level covariates, the proportion of buildings built before 1949 (Buildings1949) tends to exert negative impacts on land prices in both models. This is partly because the variable Buildings1949 is a proxy for the amount of old and low-quality housing stocks with poor living facilities. Also, the real estate developers have to incur huge removal cost when demolishing these old buildings for new housing projects. Crime rates and population density are not significantly associated with land prices in Beijing.

As for the magnitude of covariate effects, we should interpret them in terms of total, direct and indirect impacts [18]. Table 3 summarises these impact measures for each covariate supplemented with the estimated regression coefficients from the proposed method and from the classic MLM. The total or marginal impacts of each independent variable are larger than their coefficients due to a positive spatial interaction effect. As for interpreting the magnitude of these impacts, taking the example of the covariate effect of LogDcbd, if the proximity to CBD increases (or the distance to CBD decreases) by 1%, land prices will increase by 0.363% from a direct effect, and by 0.075% from an indirect effect, producing a total effect of 0.439% increase. Evaluating at the mean proximity to subway stations and the mean land price, the marginal value of decreasing the distance to nearest stations by 100 metres yields a 34.5 RMB total increase for per square metre land, which consists of a 27.8 RMB increase from direct impacts and 6.7 RMB increase from indirect impacts.

**Table 3:** The total, direct and indirect impacts of selected covariates using estimates from the proposed methodology.

MLM with spatial interaction effects						Classic MLM
	Posterior mean	Std. Error	2.5%	97.5%	Regression coefficients	Regression coefficients
***Total impacts***						
Logarea	-0.028	0.022	-0.071	0.017	-0.023	-0.031
LogDcbd	-0.439*	0.124	-0.694	-0.204	-0.362*	-0.373*
LogDsubway	-0.215*	0.051	-0.317	-0.115	-0.177*	-0.198*
LogDele	-0.018	0.048	-0.113	0.075	-0.015	-0.054
LogDpark	-0.180*	0.074	-0.324	-0.038	-0.148*	-0.245*
LogDriver	0.120*	0.043	0.037	0.202	0.099*	0.137*
Popden	0.023	0.016	-0.009	0.054	0.019	0.029*
Buildings1949	-1.313*	0.630	-2.497	-0.056	-1.082*	-1.380*
Crimerate	0.001	0.010	-0.018	0.020	0.001	0.010
***Direct impacts***						
Logarea	-0.023	0.018	-0.059	0.014	-0.023	-0.031
LogDcbd	-0.363*	0.102	-0.578	-0.168	-0.362*	-0.373*
LogDsubway	-0.178*	0.042	-0.259	-0.097	-0.177*	-0.198*
LogDele	-0.015	0.039	-0.091	0.062	-0.015	-0.054
LogDpark	-0.149*	0.061	-0.266	-0.031	-0.148*	-0.245*
LogDriver	0.099*	0.036	0.031	0.169	0.099*	0.137*
Popden	0.019	0.013	-0.007	0.044	0.019	0.029*
Buildings1949	-1.086*	0.519	-2.071	-0.050	-1.082*	-1.380*
Crimerate	0.001	0.008	-0.015	0.017	0.001	0.010
***Indirect impacts***						
Logarea	-0.005	0.004	-0.013	0.003	-0.023	-0.031
LogDcbd	-0.075*	0.028	-0.138	-0.030	-0.362*	-0.373*
LogDsubway	-0.037*	0.013	-0.066	-0.016	-0.177*	-0.198v
LogDele	-0.003	0.008	-0.021	0.013	-0.015	-0.054
LogDpark	-0.031*	0.015	-0.065	-0.006	-0.148*	-0.245*
LogDriver	0.020*	0.009	0.006	0.039	0.099*	0.137*
Popden	0.004	0.003	-0.001	0.011	0.019	0.029*
Buildings1949	-0.227*	0.125	-0.491	-0.009	-1.082*	-1.380*
Crimerate	0.000	0.002	-0.003	0.004	0.001	0.010

The last two columns are the regression coefficients from the proposed method and from the classic MLM.

* denotes statistically significant at 95% credible level or above.

#### Contextual effects visualization

**Fig 8:**
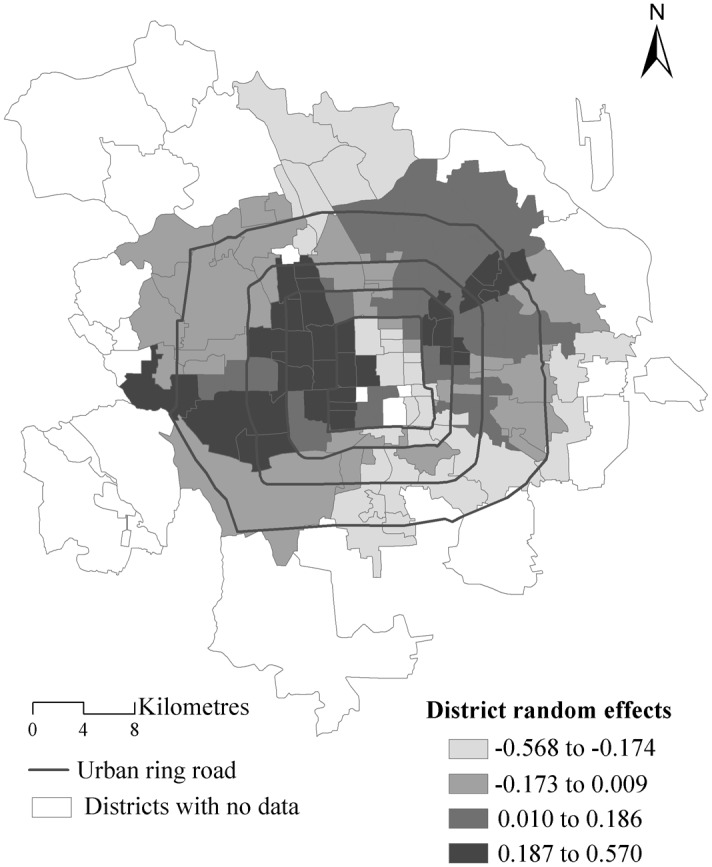
The district level random effects from the proposed MLM with spatial interactions.

Fig 8 maps the estimated posterior means of district level random effects from the proposed model. The breaking points correspond to the lower, median and upper quartiles of the district effects, with darker colours indicating stronger effects. Overall, there is a clear spatial pattern: high and low values of district effects clustering together respectively because of the significant and relatively large spatial autoregressive parameter observed (*λ*).

From the map we can identify two main areas with large district effects. These are in the northeast and the western parts of urban areas in Beijing, which is in accordance with previous studies [24]. The northeastern area has been planned as a major urban sub-centre of Beijing and has a lot of large residential communities provided with sufficient supplementary commercial facilities such as large shopping malls and with many service-related job opportunities. The land use mix improves land values especially within the large residence-function orientated urban areas [26]. For the western areas (between the second and fourth ring roads), the clustering of high land prices might be related to the concentration of jobs and educational facilities such as universities, high-quality primary and high schools.

**Fig 9:**
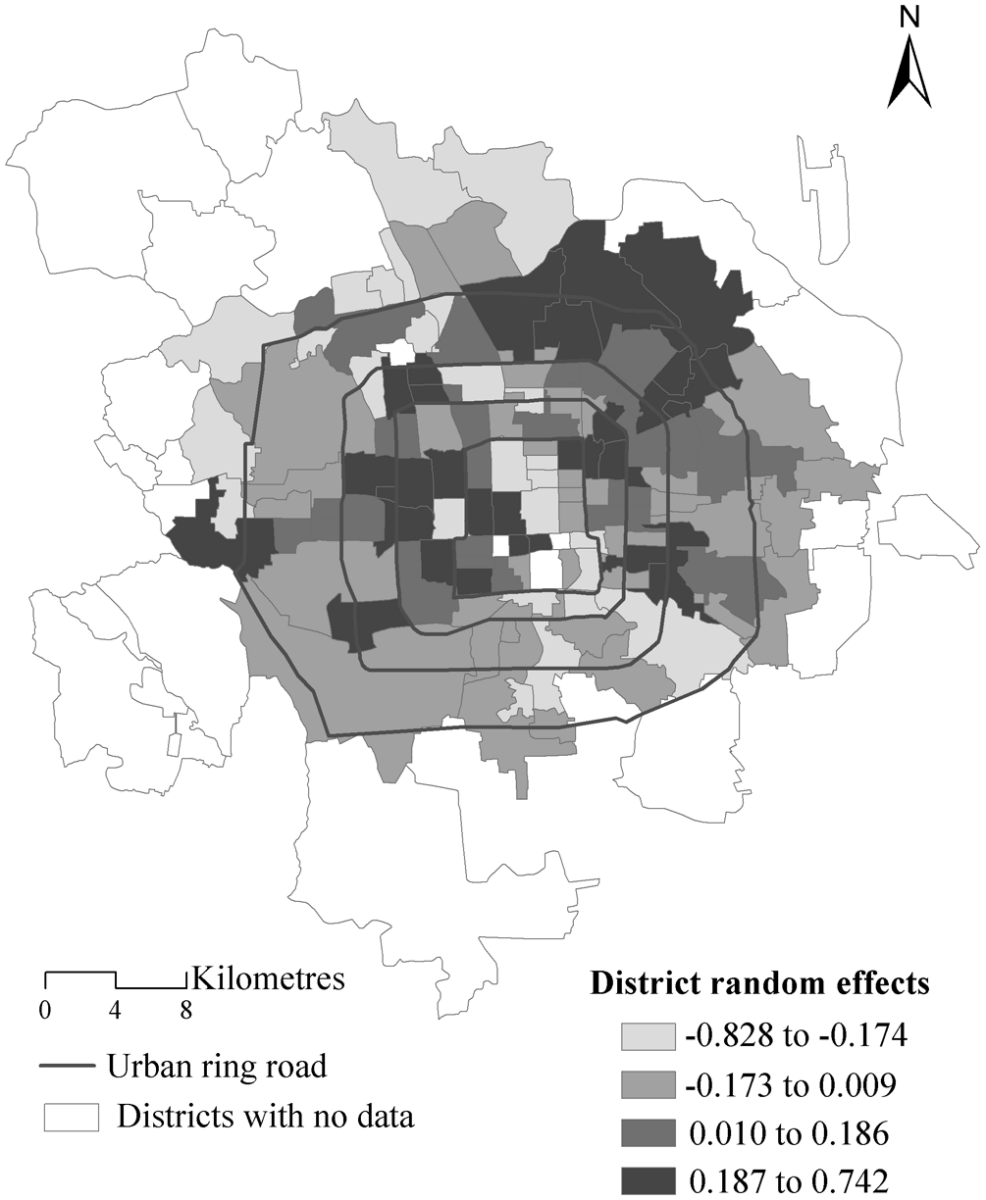
The district level random effects from the classic MLM.

For comparison, Fig 9 maps the estimated posterior means of the district level random effects from the MLM, using the same breakpoints as in Fig 8. The overall spatial pattern is more discrete than that in Fig 8 because the MLM assumes these district random effects to be independent of each other. In contrast, the proposed MLM with spatial interaction effects exploits the estimation of the random effects from the neighbouring districts to calculate the summary for a particular district, thus providing more smoothed estimates than the MLM. In addition, we test whether the district effects from the MLM are spatially dependent by using the Moran’s I statistic based on the spatial weights matrix *M* [15]. The resultant Moran coefficient is 0.223 with a p-value equal to <0.001. This demonstrates the existence of positive spatial dependence in the estimated district effects from the MLM. It contradicts the core model assumption of independence of the higher level residuals.

## Conclusions

With the increasing availability of geographically hierarchical datasets, multilevel models are widely employed to examine the outcomes of interest measured at the lower and higher levels simultaneously. The purpose is to avoid the "ecological fallacy" when transferring relationships between variables at aggregate spatial scales to individuals, to avoid the "atomistic fallacy" where correlations between variables are investigated exclusively at the individual level without consideration to the context, to look for and to quantify contextual effects, and to provide better estimates of model parameters and their standard errors in the presence of such effects [27].

Despite being frequently applied to geographical data, the classic MLM is not really a spatial modelling technique as it does not consider the possibility of proximity effects between the units of analysis. It does not model spatial interaction effects and is incapable of distinguishing between contextual effects and spatial interaction effects. The consequence associated with this is that the contextual effects estimated by employing the MLM will be confounded with any spatial interaction effects. From the simulation study, where the data generating process consists of both the contextual effects and the positive spatial interaction effects at each spatial scale, we see that the MLM produces biased estimation for the covariate effects and for the two variance parameters. The implication for future empirical studies in which the classic MLM is chosen concerns the validity of the statistical inference on covariate effects especially for the estimated contextual effects, and of the interpretation on how important group dependence is in explaining the outcome variations of interest.

This paper provides an integrated spatial econometrics and multilevel methodological framework for modelling spatial data with a hierarchical structure, allowing for separation of the (horizontal) spatial interaction effect from the (vertical) group dependence effect. Using the proposed MLM with spatial interaction effects, we find significant spatial interactions among the residential land parcels and among the districts in Beijing, China. Given the proliferation of hierarchical spatial data and their extensive use in regional economics, health and environmental research, we anticipate that our approach could be useful in a wide range of applications. Though the method is illustrated using spatial data, the approach is also suitable to model hierarchical social network data where social network effects and contextual effects might simultaneously impact outcomes or behaviours of individuals [28].

We end this paper by discussing further elaborations to the proposed MLM with spatial interaction effects. In a way similar to a random slope multilevel model, a future model extension would be to allow for spatially varying regression coefficients across space. For example, regression coefficients for certain land parcel level covariates could vary across districts. This enables us to explore spatial heterogeneity in the covariate effects. Spatial heterogeneity in the covariate effects across higher level spatial units can be constructed by using a multivariate conditional autoregressive process [20,29]. This extension is our next step towards an integrated spatial and multilevel modelling technique.

## Supporting Information

S1 Appendix R code to implement the developed methodology. We provide the R code to implement the developed methodology. In addition, R scripts that demonstrate how to use the core function and reproduce the land price model results in the manuscript are provided.
(ZIP)

S1 Dataset The land price data set used in the empirical study. (CSV)
